# Urinary Microbiome in Bladder Diseases—Review

**DOI:** 10.3390/biomedicines11102816

**Published:** 2023-10-17

**Authors:** Joanna Chorbińska, Wojciech Krajewski, Łukasz Nowak, Bartosz Małkiewicz, Francesco Del Giudice, Tomasz Szydełko

**Affiliations:** 1 Department of Minimally Invasive and Robotic Urology, University Center of Excellence in Urology, Wrocław Medical University, 50-367 Wrocław, Poland; wojciech.krajewski@umw.edu.pl (W.K.); lllukasz.nowak@gmail.com (Ł.N.); bartosz.malkiewicz@umw.edu.pl (B.M.); 2 Department of Maternal Infant and Urologic Sciences, Sapienza University of Rome, Policlinico Umberto I, 00161 Rome, Italy; francesco.delgiudice@uniroma1.it; 3 University Center of Excellence in Urology, Wrocław Medical University, 50-367 Wrocław, Poland; tomasz.szydelko@umw.edu.pl

**Keywords:** urinary microbiome, urobiome, bladder cancer, urinary tract infection, urinary incontinence, overactive bladder, neuropathic bladder, bladder pain syndrome

## Abstract

The microbiome is the totality of microorganisms found in a specific biological niche. It has been proven that in the human body, the microbiome is responsible for its proper functioning. Dysbiosis, i.e., a disturbance in the composition of the microbiome, may be associated with the pathogenesis of many human diseases. Until recently, studies did not focus on the microbiome of the urinary tract, because, since the 19th century, there had been a dogma that urine in healthy people is sterile. Yet, advances in molecular biology techniques have allowed this dogma to be overthrown. The use of DNA sequencing has shown that the urinary tract has its own endogenous microbiome. This discovery enabled further research on the characteristics of the urine microbiomes of healthy people, as well as on the role of the urine microbiome in the pathogenesis of many urological diseases, including bladder diseases. The aim of this review is to summarize the current knowledge on the urinary microbiome in bladder diseases and to identify potential directions for further research.

## 1. Introduction

The ‘microbiota’ is defined as the totality of microorganisms living in a specific environment, and the term ‘microbiome’ refers to the totality of microbial genes in a specific biological niche. However, in the literature these terms are often used interchangeably [1].

Issues related to the human microbiome and its impact on human functioning and the pathophysiology of various diseases are widely studied. A new direction of research on the microbiome is the analysis of the urobiome—the urinary tract microbiome and the relationship between its composition and the pathogenesis of many urological diseases. This is due to the fact that the dogma regarding the sterility of urine in healthy people has only recently been overthrown. It was proposed in the 19th century by Louis Pasteur and has been in use for many years. Only the progress in DNA sequencing made it possible in 2011 to detect the urobiomes of healthy people. The use of sequencing of the 16S ribosomal RNA (rRNA) gene allowed the detection of bacteria in the urine that are not identifiable by standard urine culture. This led to the overthrow of the dogma and made it possible to start research on the urinary microbiome [2].

The 16S rRNA gene encodes the 16S rRNA, forming the 30S subunit of the prokaryote ribosome. The use of this gene results from its structure; it consists of evolutionarily conservative regions and hypervariable regions. Conserved regions are present in all bacteria and are used to design sequencing primers, while hypervariable regions (V1-V9) vary between species. Their comparison with databases allows the taxonomic classification of bacteria [3].

**Figure 1:**
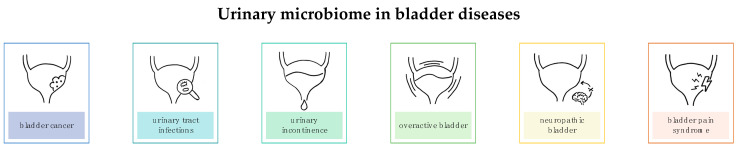
Urinary microbiome in bladder diseases.

The aim of this review is to summarize the current knowledge on the urinary microbiome in bladder diseases and to identify potential directions for further research (Figure 1).

## 2. Evidence Acquisition

A literature search was performed using the PubMed electronic databases. The search was limited to articles published until January 2023. Searched terms included: “urinary microbiome”, “urobiome”, “bladder cancer”, “urinary tract infection”, “acute cystitis”, “recurrent urinary tract infection”, “urinary incontinence”, “stress urinary incontinence”, “urgency urinary incontinence”, “mixed urinary incontinence”, “overactive bladder”, “neurogenic bladder”, “neuropathic bladder”, “bladder pain syndrome”, “painful bladder syndrome” and “interstitial cystitis”. Boolean operators (NOT, AND, OR) were used in succession to narrow and broaden the search. Only publications in English were considered and evidence was limited to human data. The review mainly included papers that used 16S rRNA sequencing to identify the bacteria. Only in exceptional cases were single papers based on expanded quantitative urine culture included.

The references of the relevant review articles were also manually screened to ensure that no additional eligible papers were inadvertently omitted. Additional screening was also performed on ahead-of-print articles published in various urological journals.

## 3. Urinary Microbiome

The urinary microbiome of healthy people is less abundant and less diverse compared to the microbiome of other body niches. Its composition depends on many factors, such as gender, age, lifestyle, medications and supplements used, diet, hormonal status and the presence of infections.

Overall, the urinary microbiome does not differ significantly between healthy women and men, but differences in the abundance of various genera of bacteria can be observed between the sexes. The most common genera of bacteria found in the urinary microbiomes of healthy women and men include *Prevotella*, *Escherichia*, *Enterococcus*, *Streptococcus*, *Staphylococcus*, *Corynebacterium*, *Lactobacillus* and *Citrobacter*. The differences between the sexes are slight. The genus *Lactobacillus* is more abundant in the urine microbiomes of healthy women, while the genera *Corynebacterium* and *Streptococcus* dominate in the microbiomes of healthy men [4,5,6].

Not only gender, but also age influences the composition of the urinary microbiome, but the available data are small and concern mainly women. Although no differences in microbiome diversity depending on age were found, a decrease in the relative abundance of the *Lactobacillus* genus was observed in older women and an increase in the abundance of the genera *Mobiluncus*, *Oligella* or *Porphyromonas* was observed in postmenopausal women [7]. 

However, due to the small number of studies and small study groups, further research is necessary, and conclusions should be drawn with caution.

A summary of the composition of the urinary microbiome in bladder diseases is presented in Table 1. 

## 4. Bladder Cancer (BC)

Bladder cancer (BC) is one of the most common cancers of the urinary system and is the tenth most frequently diagnosed malignancy in the general population, and the seventh in the male population. Many risk factors for bladder cancer have been identified, and smoking is considered the most important of them. Others include occupational exposure to aromatic amines, polycyclic aromatic hydrocarbons and chlorinated hydrocarbons, or a history of pelvic radiotherapy [27]. It is believed that the urinary microbiome may also play a role in the pathogenesis of BC.

A summary of the urinary microbiome composition in BC is presented in Table 2.

Many attempts have been made to characterize the urinary microbiomes of patients with bladder cancer. Already in 2015, Popovic et al. compared the urinary microbiomes of 12 BC men with 11 controls. In both groups, the urine for analysis was collected through clean catch mid-stream urine (MSU). The study found no differences in diversity between the groups; however, *Fusobacterium*, *Actinobaculum*, *Facklamia* and *Campylobacter* genera were more common in the urine of BC patients [8]. Another study by Xu et al. found a high incidence of *Streptococcus, Pseudomonas* and *Anaerococcus* genera in BC patients’ urobiomes. They compared MSU samples from eight BC patients and six healthy controls [9]. Contrary to Popovic et al., Hussein et al. found differences in urobiome beta diversity between BC patients and healthy controls. They analyzed the urinary microbiomes of 43 BC patients and 10 healthy controls. Urine for analysis from healthy individuals was collected through MSU, and from patients with BC transurethral during cystoscopy, transurethral resection of the bladder tumor (TURB) or cystectomy, or less often through MSU. They revealed that the genera *Actinomyces*, *Achromobacter*, *Brevibacterium* and *Brucella* were enriched in the urine of BC patients [10]. However, the limitation of the study was the high heterogeneity of the methods of collecting urine for analysis, which could significantly affect the obtained results. Similar results were obtained by Bi et al., showing a greater diversity of the urinary microbiomes in patients with BC, and also a higher prevalence of the *Actinomyces* genus, especially *Actinomyces europaeus* [38]. More recent studies do not clearly confirm previously obtained results. Mai et al. compared the MSU microbiomes of 24 BC patients with the urinary microbiomes of healthy individuals obtained from samples from other laboratories. They found greater abundance of *Acinetobacter*, *Proteobacteria*, *Firmicutes*, *Actinobacteria*, *Bacteroidetes*, and *Tenericutes* in the urine of BC patients [11]. A more frequent occurrence of the genus *Acinetobacter* was also shown by Wu et al. They compared the urinary microbiomes of 31 BC men with the urinary microbiomes of 18 healthy controls. In both groups, the urine for analysis was collected through MSU. In addition to the genus *Acinetobacter*, an enrichment of the genera *Anaerococcus*, and *Sphingobacterium* and a decrease in the genera *Serratia*, *Proteus* and *Roseomonas* were observed in the urobiomes of BC patients. Moreover, they noticed a greater richness of urinary microbiomes in patients with bladder cancer [12]. Opposite results were obtained by Chipollini et al., who observed a lower richness and evenness of the urobiomes of patients with BC. They postulated a hypothesis in which the urinary microbiome is rich in healthy people, while in the neoplastic process one microbial community dominates [32]. Unfortunately, so far, all studies assessing the relationship between bladder cancer and the urobiome are based on small samples, and the conclusions should be treated with caution. Further multicenter studies on a large patient population are necessary. Moreover, the diagnosis of bladder cancer is based on invasive methods such as cystoscopy or TURB and for now, according to the EAU guidelines, it cannot be replaced by any other non-invasive test, including urine testing [27]. Therefore, it seems that the role of urine microbiome assessment in the diagnosis of bladder cancer will be limited or supportive.

It is believed that the different incidences of BC between sexes may result not only from differences in the prevalence of smoking between females and males, as was believed for many years, but also from the influence of other factors, such as different composition of the urinary microbiome between sexes. As mentioned earlier, Hussein et al. analyzed the urobiomes of 43 BC patients collected transurethrally during cystoscopy, TURB, cystectomy or less commonly by MSU. They compared urine samples from seven women and thirty-six men with BC. There were no significant differences in the diversity of the urinary microbiome between men and women; however, the urine of females with BC was enriched in the genera *Lactobacillus*, *Actinotignum*, *Prevotella*, *Veillonella* and *Campylobacter* [10]. A similar analysis was performed by Pederzoli et al., who compared the urinary microbiomes of 49 BC patients qualified for radical cystectomy with the urinary microbiomes of 59 healthy controls. They revealed that the genus *Klebsiella* was more common in the urine of BC females [28]. Due to the small amount of data, further research is needed in this regard.

Smoking is the most important risk factor for developing BC. The main pathomechanism consists in the accumulation of aromatic amines excreted in the urine and polycyclic aromatic hydrocarbons contained in tobacco smoke. In the phase of urine accumulation in the bladder, they affect the urothelial epithelial cells, leading to damage to their DNA and the development of cancer. It is suspected that smoking may lead to a change in the composition of the urine microbiome and, consequently, to the development or increased incidence of BC [27]. In order to assess the effect of smoking on the composition of the urobiome, Ma et al. compared the urine of 15 BC males and 11 healthy controls. Urine was collected by MSU and both groups were subgrouped according to smoking status. The study found differences in alpha and beta diversity between smokers and non-smokers, with higher alpha diversity in BC smokers. In addition, the urinary microbiomes of smokers were enriched in *Bacteroidaceae*, *Erysipelotrichales*, *Lachnospiraceae* and *Bacteroides* [29]. Opposite results were obtained by Moynihan et al., who assessed the microbiome of urine collected by MSU from 43 men with BC and hematuria. They found no differences in alpha and beta diversity between smokers and non-smokers [39]. 

Depending on the stage of the cancer, we distinguish non-muscle-invasive BC (NMIBC) and muscle-invasive BC (MIBC). It is postulated that the composition of the microbiome differs depending on the cancer stage, and also, in the NMIBC group, depending on the risk of progression and recurrence, which in the future could be used as a non-invasive biomarker for stratifying the risk of progression and recurrence. In their studies, Zeng et al. and Qiu et al. found greater alpha diversity in the group of patients with recurrence of NMIBC. Zeng et al. analyzed the urinary microbiomes of 40 BC patients who were followed up for 12 months. They found an increased presence of *Anoxybacillus*, *Massilia*, *Thermomonas*, *Brachybacterium*, *Micrococcus*, *Nocardioides*, *Larkinella*, *Jeotgalibacillus* and *Geomicrobium* in the urine of the recurrence group [30]. Qiu et al. assessed the urine microbiomes of 40 men with BC, including 12 with recurrence and 28 without it. They revealed that the urinary microbiomes of the recurrence group was enriched with *Pseudomonas*, *Staphylococcus*, *Corynebacterium* and *Acinetobacter* genera [31]. Wu et al. compared the urine microbiomes collected by MSU from 31 men with BC according to the risk of progression and recurrence based on the EORCT scoring model. They noted that the genera *Herbaspirillum*, *Porphyrobacter*, *Bacteroides* and *Marmoricola* were overrepresented in the group of high risk of progression and the genera *Herbaspirillum*, *Gemella*, *Bacteroides*, *Porphyrobacter*, *Faecalibacterium* and *Aeromonas* in the group with high risk of recurrence [12]. Comparing the urine microbiomes of patients with NMIBC and MIBC, Hussein et al. found that the *Hemophilus* and *Veillonella* genera were significantly more abundant in the urine of MIBC patients, while the *Cupriavidus* genus was significantly more abundant in the urine of NMIBC patients. As previously mentioned, they analyzed transurethral urine collected by cystoscopy, TURB or cystectomy from 43 patients with BC, including 29 with NMIBC and 14 with MIBC [10]. Chipollini et al. however, evaluated urine collected by the MSU from 27 BC patients, including 12 NMIBC and 15 MIBC, and revealed a higher prevalence of *Bacteroides* and *Faecalibacterium* genera in the urine of MIBC patients [32]. Again, the studies were carried out on small samples, and the methods of collecting urine samples for analysis were very diverse. Additionally, the available number of studies comparing patients with NMIBC and MIBC is very small. All these factors lead to heterogeneity of the results obtained and affect their reliability. The results should be considered as preliminary, and the conclusions should be treated with caution.

The exact mechanism of the influence of the urinary microbiome on NMIBC recurrence is not yet understood. It is believed that the interaction of the urobiome and the programmed cell death protein ligand 1 (PD-L1) pathway may be responsible for this. This pathway is one of the tumor immune escape mechanisms and is also present in BC. Chen et al. hypothesized that the urinary microbiome may be involved in the recurrence and progression of NMIBC by up-regulating PD-L1 expression. Therefore, they analyzed the urinary microbiomes collected by MSU from 28 men with NMIBC. The subjects were divided into a PD-L1-positive group and a PD-L1-negative group based on PD-L1 immunohistochemical results. The authors discovered a greater richness of the urinary microbiomes in the PD-L1-positive group. In addition, in this group’s urobiomes, enrichment of the genera *Leptotrichia*, *Roseomonas* and *Propionibacterium* and a decrease in genera *Prevotella* and *Massilia* were demonstrated. The authors postulate the possible involvement of the urinary microbiome in the expression of PD-L1 [33].

Currently, there are no clear guidelines on how to collect urine for microbiome analysis. The authors most often use MSU or transurethral catheterization (TC). Urine collection by MSU leads to the presence of the urethral microbiome in the sample and carries the risk of contamination of the sample with skin bacteria; however, the use of TC in healthy individuals is ethically questionable due to the risk of catheter-related complications. To clarify these doubts, Hourigan et al. compared the microbiomes of urine collected from the same patients by MSU and TC. Twenty-two patients with BC were analyzed, including eight females and fourteen males. There were no significant differences in alpha and beta diversity across samples between urine collection methods. However, the analysis by sex revealed differences between the collection methods in the male group. The authors recommended the use of only one urine collection method per examination [37].

More and more research is devoted to the role of the tumor microenvironment and the role of the tumor microbiome in the creation and functioning of this microenvironment. Liu et al. were the first to analyze the tissue microbiomes of BC patients. They compared 22 samples of cancer tissue with 12 samples of healthy tissue located about 5 cm from the cancer tissue. They found lower alpha and beta diversity in tumor tissue and higher abundance of *Cupriavidus* spp., an unknown genus of the family *Brucellaceae*, and *Acinetobacter*, *Anoxybacillus*, *Escherichia-Shigella*, *Geobacillus*, *Pelomonas*, *Ralstonia* and *Sphingomonas* in cancer samples [34]. Similar results were obtained by Parra Grande et al., who assessed the tissue microbiomes of 52 paired samples (matched tumor and non-tumor samples) from 26 BC patients. They also found lower microbiome alpha diversity in cancer tissue, and they additionally found lower microbiome richness in this group. In tumor tissue, the most frequent phyla were *Firmicutes* and *Bacteroidetes*, followed by *Proteobacteria* and *Actinobacteria*, representing 98.53% of the total microbiota. At the genus level, the most frequent genera were *Bacteroides*, *Escherichia-Shigella*, *Staphylococcus* and *Enterococcus*, representing 31.99% of the total microbiota [35]. In another study, Mansour et al. compared the composition of the tumor tissue microbiome with the urine microbiomes collected from the same set of 10 BC patients during TURB. They revealed an overrepresentation of five genera in tissue samples compared to urine. These were *Akkermansia*, *Bacteroides*, *Clostridium sensu stricto*, *Enterobacter* and *Klebsiella* [36]. Further research on this topic will improve the accuracy of diagnosing disorders using the composition of the microbiome.

The role of the microbiome is suggested not only in the incidence, progression and recurrence of BC, but also in response to treatment. Bacillus Calmette-Guérin (BCG) intravesical immunotherapy is one of the methods of treatment for patients with high-risk NMIBC. In a previously mentioned study, Hussein et al. also compared the urine microbiomes of 11 NMIBC patients undergoing BCG therapy. They found that the genera *Serratia*, *Brochothrix*, *Negativicoccus*, *Escherichia-Shigella* and *Pseudomonas* were more common in the urine microbiomes of patients who responded to BCG therapy compared to those who did not [10]. Sweis et al. also analyzed the urine microbiomes of patients undergoing BCG therapy. They compared the urine microbiomes collected through TC from 31 patients with NMIBC who received BCG instillations. The abundance of *Proteobacteria*, especially *Gammaproteobacteria*, was higher in patients undergoing BCG therapy with recurrence. *Firmicutes*, such as *Lactobacillales*, were more abundant in patients without recurrence [40]. 

The mechanism by which the urinary microbiome influences the response to intravesical BCG instillations is not fully understood, but it is believed that it may be due to the influence of the urobiome on the mucosal defensin levels. There are three types of defensins, induced human beta defensin 2 (HBD2), induced human beta defensin 3 (HBD3) and constitutively produced human beta defensin 1 (HBD1). HBD1 has a protective effect against the development of BC. What is more, the urobiome may be responsible for the lack of response to BCG therapy through its effect on the levels of HBD2 and 3. Mansour et al. assessed the relationship between the tissue and urinary microbiomes, HBD expression and HBD urine levels in BC patients. They compared tissue and urine samples from 55 BC patients with urine samples collected by MSU from 35 healthy controls. In patients with BC, HBD1 levels were reduced and HBD2 and 3 levels were increased. *Staphylococcus*, *Corynebacterium* and *Oxyphotobacteria* genera were significantly more common in the urinary microbiomes of BC patients, and *Faecalibacterium* and *Bacteroides* genera were significantly less frequent. Analysis of the relationship between the microbiome composition and the levels of defensins found that the abundance of the genera *Bacteroides*, *Parabacteroides* and *Faecalibacterium* gradually decreased as the levels of HBD2 and HBD3 increased. In addition, a higher abundance of *Corynebacterium* and *Staphylococcus* was noted, along with higher HBD2 and 3 urinary levels [41]. To date, the data are insufficient to clearly determine whether the urinary microbiome influences the response to BCG therapy. Further research is necessary not only to confirm this relationship, but also to demonstrate the exact mechanism responsible for it.

## 5. Urinary Tract Infections (UTIs)

A summary of the composition of the urinary microbiome in UTIs is presented in Table 3.

### 5.1. Acute Cystitis (AC)

Acute cystitis (AC) is an infection of the bladder with symptoms such as dysuria, pollakiuria and urgency. In non-pregnant women without anatomic or functional abnormalities of the urinary system, acute cystitis is defined as uncomplicated, otherwise it is defined as complicated [44].

Ceprnja et al. conducted an analysis of the urinary microbiomes of 28 patients with AC, including 16 women and 12 men, in which urine was collected both through MSU and TC. They found that the classes *Gammaproteobacteria* and *Bacilli* dominated all samples, with *Proteobacteria*, *Firmicutes*, *Bacteroidetes* and *Actinobacteria* accounting for more than 99% of all detected taxa. In addition, in most cases, dominance of a single family was observed, in which the families *Enterococcaceae*, *Enterobacteriaceae* or *Pseudomonadaceae* accounted for more than 90% of the total bacterial abundance [13]. Similar results were obtained by Yoo et al., who compared the microbiomes of urine collected by TC between 11 women with AC and 31 women with recurrent urinary tract infection (rUTI). As in the case of Ceprnja et al., they found a higher prevalence of the class *Gammaproteobacteria* and the phylum *Proteobacteria* in patients with AC, compared to patients with rUTIs, particularly genera *Pseudomonas* and *Acinetobacter* and family *Enterobacteriaceae* [14].

It is believed that the urinary microbiomes in patients with AC vary according to sex and age. Willner et al. assessed the MSU urinary microbiomes from 50 patients with AC and found that *Enterobacteriaceae*, in particular *E. coli*, were more common in younger patients, while *Pseudomonas* and the phylum *Firmicutes* were more abundant in older patients. In addition, they detected a high abundance of *Enterobacteriaceae* and the presence of lactic acid bacteria and other *Bacilli* in the urine of females, but the genera *Streptococcus*, *Lactobacillus* and *Staphylococcus* were not detected in any males [42].

Some patients, after urogynecological surgery, develop a urinary tract infection [UTI]. Nienhouse et al. and Thomas-White et al. assessed the relationship between the urinary microbiomes of patients before urogynecological surgery and the risk of a UTI in the postoperative period. In both studies, urine was collected by TC on the day of surgery. Nienhouse et al. evaluated the urinary microbiomes of 54 women and postulated that a more diverse urinary microbiome has a protective effect against the development of UTIs postoperatively, as opposed to a microbiome dominated by a single type [45]. Thomas-White et al. analyzed the urinary microbiomes of 104 women and found that the risk of UTI was related to the depletion of *Lactobacillus iners* and the enrichment of a diverse mixture of uropathogens. They believe that the abundance of *L. iners* has a protective effect against the development of UTIs in the postoperative period [46]. 

It is believed that the dynamics of changes in the urinary microbiome during AC treatment may, in the future, lead to the determination of the recommended duration of antibiotic therapy. Ceprnja et al. analyzed changes in the urinary microbiome in one patient with AC, caused by *K. pneumoniae* and treated with 1 g of cephalexin orally for seven days. Eight urine samples, collected at 24-h intervals starting before antibiotic therapy, were analyzed. Before treatment, the family *Enterobacteriaceae* dominated in the urinary microbiome, which accounted for over 95% of all detected bacteria, but after 24 h from the first dose of the antibiotic, it was reduced to 1.28% of all detected bacteria. A gradual increase in the number of the families *Lactobacillaceae* and *Pseudomonadaceae* was observed on subsequent days, with *Lactobacillaceae* dominating until the 7th day of the study and *Pseudomonadaceae* dominating on the last day of treatment. Microbiome diversity was lower on days 3 and 4 of the study with an increase on days 5 and 6. Gram-negative bacteria declined steadily on days 1 to 4, followed by an increase on days 5 and 6. *K. pneumoniae* was shown to be responsive to treatment; however, continued antibiotic therapy after 3 days had a significant effect on the host’s urinary microbiome, making the urinary tract vulnerable to reinfection. This allowed the opportunistic pathogens from the family *Pseudomonadaceae* to multiply, which in turn led to the recurrence of AC symptoms in the examined patient. Due to the fact that the analysis concerned only one patient, further research is needed to be able to draw any conclusions [13].

Various methods of preventing UTIs are constantly being sought. It is believed that oral probiotics may reduce the risk of UTIs. Wolff et al. assessed the effect of oral probiotic supplementation (*Lactobacillus rhamnosus* GR-1 and *Lactobacillus reuteri* RC-14) on the uropathogens/*Lactobacillus* (U/L) ratio in the urine of healthy premenopausal women. It was a randomized, double-blind, placebo-controlled trial in which seven patients were enrolled (four in the probiotic group and four in the placebo group). The patients collected urine through MSU every day for 3 months, and in the placebo group they took probiotics on days 21–60. The placebo and probiotic groups had similar mean U/L ratios, with no difference between the placebo and probiotic groups. The probiotic species were never identified in the voided urine. There were no changes between groups in terms of microbiota diversity. However, bacterial identification was obtained by expanded quantitative urine culture, not by 16S rRNA gene sequencing. It seems that oral use of probiotics does not play a role in the prevention of UTIs, but further research on a larger number of patients is necessary [47].

### 5.2. Recurrent Urinary Tract Infection (rUTI)

According to the EAU guidelines, a recurrent urinary tract infection (rUTI) is defined as a minimum of three UTIs per year or two UTIs in the last 6 months. This applies to both complicated and uncomplicated infections [44].

To assess the relationship between the urinary microbiome and rUTIs, Huang et al. compared the urinary microbiomes of 90 rUTI women with the urinary microbiomes of 62 healthy controls. In both groups, urine was collected by MSU for analysis. They found that the abundance of *Proteobacteria*, *Burkholderiales*, *Ralstonia*, *Prevotella*, *Dialister* and *Corynebacterium* was higher in patients with rUTI compared to healthy subjects, suggesting that different species other than *Enterobacteriaceae* may be responsible for rUTIs. Moreover, the abundance of *Lactobacillus*, *Gardnerella*, *Viridans streptococci* and *Ezakiella* was significantly reduced in patients with rUTI compared to healthy controls. In addition, the urine diversity of rUTI patients was lower than that of healthy controls, supporting the hypothesis that a less diverse microbiome favors infection and a more diverse microbiome is protective [15]. Similar conclusions regarding the role of species other than *Enterobacteriaceae* in the pathogenesis of rUTI were reached by Yoo et al. As mentioned earlier, they compared the urinary microbiomes of 11 women with AC and 31 women with rUTI. They found that alpha diversity and richness were significantly higher in patients with rUTI compared to AC. The urinary microbiomes of the rUTI group had a higher abundance of the phylum *Bacteroidetes*, class *Bacteroidia*, order *Bacteroidales*, family *Prevotellaceae* and phylum *Firmicutes*. In addition, *Sphingomonas*, *Staphylococcus*, *Streptococcus* and *Rothia* spp. were detected in the urine of patients with rUTI [14].

rUTI is more common in postmenopausal women. It is believed that postmenopausal hormonal changes lead to a change in the composition of the microbiomes of both the vagina and the bladder, which leads to a greater susceptibility to urinary tract infections. To prove this, Hugenholtz et al. compared the urinary microbiomes of rUTI patients and healthy controls in both premenopausal and postmenopausal periods. The premenopausal group consisted of 18 rUTI patients and 18 healthy controls, and the postmenopausal group consisted of 20 rUTI patients and 30 healthy controls. However, the study did not show statistically significant differences in the mean relative bacterial counts between premenopausal and postmenopausal patients, depending on rUTI status [48].

Local estrogen therapy used in the postmenopausal period has been shown to reduce the risk of rUTI. It is suspected that this may be related to changes in the urinary microbiome. Angliom et al. compared the urinary microbiomes of 17 rUTI patients with the urinary microbiomes of 20 healthy controls after 3–6 months of topical estrogen therapy. All women were in the postmenopausal period and their urine was collected for analysis by TC. The authors found that topical estrogen therapy leads to a decrease in the *Finegoldia magna* abundance in the urinary microbiome, which may be protective against the development of rUTI. However, there was no change in the relative abundance of *Lactobacillus* spp. after estrogen therapy, in both the rUTI and control groups [49]. Different results were obtained by Jung et al., who analyzed the urinary microbiomes collected by MSU from 17 patients using various forms of topical estrogen therapy (conjugated estrogen cream, estradiol ring) for 6 months. They revealed that the relative abundance of *Lactobacillus crispatus* significantly increased after estrogen treatment, which may be responsible for the successful treatment of rUTI with topical estrogen therapy [43].

## 6. Urinary Incontinence

According to the International Continence Society (ICS), urinary incontinence is defined as a complaint of involuntary loss of urine. This disorder causes both hygienic and social problems and contributes to the deterioration of the quality of life. A distinction is made between stress incontinence, urgency urinary incontinence and mixed urinary incontinence [50].

A summary of the composition of the urinary microbiome in urinary incontinence is presented in Table 4.

### 6.1. Urgency Urinary Incontinence (UUI)

Urgency urinary incontinence (UUI) is the involuntary leakage of urine caused by a sudden unstoppable urge and contraction of the bladder detrusor muscle [50]. 

A summary of the composition of the urinary microbiome in UUI is presented in Table 5.

Pearce et al. found that the urinary microbiomes of UUI patients differs from the urinary microbiomes of healthy patients. They compared urine collected by TC from 60 UUI patients and 58 controls. The urobiomes of UUI patients were characterized by an increased frequency of *Gardnerella* and a decreased frequency of *Lactobacillus* compared to the controls, although these were the most common types of bacteria in both cohorts. Detailed species analysis found that *Lactobacillus gasseri* was more common in the UUI group and *Lactobacillus crispatus* in controls. In patients with UUI, nine genera (*Actinobaculum*, *Actinomyces*, *Aerococcus*, *Arthrobacter*, *Corynebacterium*, *Gardnerella*, *Oligella*, *Staphylococcus* and *Streptococcus*) were more common, and four of them (*Actinobaculum*, *Aerococcus*, *Arthrobacter* and *Oligella*) were isolated only in UUI patients. However, no significant differences in richness and diversity were found between UUI and control patients [16]. Also, Nardos et al. [53] and Karstens et al. [17] found no significant differences in the diversity of the urinary microbiomes collected by TC between patients with UUI and controls. Moreover, the dominant species in both groups was *Lactobacillus*. However, Karstens et al. demonstrated that the severity of UUI symptoms was associated with a reduced diversity in the microbiomes of women with UUI [17].

The research team of Pearce et al. also assessed the relationship between the microbiomes of urine collected by TC and sociodemographic factors in 182 women treated for UUI. They found that patients with detected bacteria genetic material were younger, had higher body weight, had a higher number of urge episodes, developed UTIs less frequently and responded better to treatment. The genera *Lactobacillus*, *Gardnerella*, *Prevotella*, *Enterobacteriaceae*, *Staphylococcus*, *Aerococcus* and *Bifidobacterium* dominated in this group, which partially coincides with the earlier work of these authors [51].

Patients with UUI, refractory to standard antimuscarinic therapy, often experience rUTI. Chen et al. monitored nine patients with refractory UUI for 2 years and analyzed 102 MSU samples. They revealed that patients with refractory UUI and coexisting rUTI possessed diverse microbiomes. They suggest that persistent bladder colonization may augment the pathology of their chronic condition [54].

Single studies investigate the effect of different treatments for UUI on the urinary microbiome. Thomas-White et al. compared the urobiomes of 74 UUI patients taking 5 mg of solifenacin, with the possibility of dose escalation to 10 mg for inadequate UUI symptom control, with 60 controls. Urine for analysis was collected by TC once in the control group and three times in the UUI group: before treatment and 4 and 12 weeks after starting treatment. The study found that the urinary microbiomes of UUI and healthy patients varied in diversity, with greater diversity in the UUI group. These results contradict those described above. In addition, response to treatment was also related to the diversity of the microbiome. Patients with a clinical response to solifenacin had a less diverse urinary microbiome, and non-responders had a more diverse microbiome, often containing bacteria not typically found in responders. However, as in the previously mentioned studies, the genus *Lactobacillus* dominated in both groups [55]. Interestingly, the opposite results were obtained by Halverson et al., who analyzed the urinary microbiomes of 47 UUI patients treated with mirabegron. Urine for analysis was obtained by TC at baseline and after 4, 8 and 12 weeks. After 12 weeks, the responders’ urinary microbiomes became significantly richer and more diverse than the non-responders’, and there was, at baseline, no significant difference in alpha diversity between these groups [56].

Analysis of the effect of sacral neuromodulation on the urinary microbiomes of 19 UUI patients by Mueller et al. found no difference in beta diversity after 3 months of treatment, despite the reduction in the severity of symptoms in all patients [57].

The role of the urinary microbiome in response to UUI treatment cannot be ruled out, but more research is needed.

### 6.2. Stress Urinary Incontinence (SUI)

Stress urinary incontinence (SUI) is the involuntary leakage of urine caused by an increase in pressure in the abdominal cavity during various activities such as laughing, coughing or exercise [50].

To assess the relationship of the urinary microbiomes of SUI patients with their sociodemographic features, Thomas-White et al. analyzed urine collected by TC or MSU from 197 women with SUI qualified for surgical treatment. They found that bacterial diversity was significantly associated with elevated BMI, increased Medical, Epidemiologic, and Social Aspects of Aging urge index score and hormonal status, but not associated with SUI symptoms. Hormone-positive patients (pre-menopausal or post-menopausal on exogenous hormones) had a higher frequency of *Lactobacillus* and *Gardnerella* compared to hormone-negative patients [52]. 

Fok et al. assessed whether the urobiome is related to urological symptoms before and after surgery in a mixed group of 126 patients with SUI and/or prolapse. Urine was collected by TC for analysis. In the preoperative period, the most common genera were *Lactobacillus*, *Corynebacterium*, *Gardnerella*, *Staphylococcus* and *Enterobacter*. The severity of symptoms before surgery was significantly worse in patients who had bacterial genetic material detected in the urine. In particular, worse severity of symptoms was significantly associated with a higher abundance of two bacterial species: *Atopobium vaginae* and *Finegoldia magna* [18]. 

### 6.3. Mixed Urinary Incontinence (MUI)

Mixed urinary incontinence (MUI) is the involuntary leakage of urine caused by stress or urgency [50].

It seems that the urinary microbiomes of MUI patients does not differ significantly from the urinary microbiomes of healthy patients. Komesu et al. compared the microbiomes of urine collected by TC from 123 MUI patients with 84 controls. The bacterial community types did not differ between MUI patients and controls. Also, overall predominance of *Lactobacillus* did not differ between the MUI and controls. However, detailed comparison of the bacterial taxa of MUI patients and controls using Dirichlet Multinomial Mixture (DMM) modeling found that controls were more likely to have a highly dominant *Lactobacillus* community while women with MUI were more likely to have communities with lower proportions of *Lactobacillus* [19]. Similar results were shown by the analysis of the urobiomes of patients with UUI and an overactive bladder (discussed below). Collectively, these studies appear to support the concept that *Lactobacillus* may be associated with the absence of urinary symptoms. The advantage of this work is the large number of analyzed patients, but more research is needed on this topic.

Richter et al. assessed whether the urinary microbiomes of MUI patients is related to the response to surgery 12 months after a midurethral sling operation. The predominant genera in patients with MUI were *Lactobacillus*, *Gardnerella*, *Tepidimonas*, *Escherichia*, *Streptococcus* and *Prevotella*. Moreover, urobiome beta diversity was significantly associated with age in both the surgical responders and non-responders. However, no significant relationship was found between the urinary microbiome and response to surgery [20].

## 7. Overactive Bladder (OAB)

Overactive bladder (OAB) is a disease defined by the International Continence Society as urinary urgency, usually accompanied by frequency and nocturia, with or without UUI, in the absence of an UTI or other obvious pathology [50].

A summary of the composition of the urinary microbiome in OAB is presented in Table 6.

A study comparing the microbiomes of midstream urine from 63 women with OAB with 35 controls by Curtiss et al. found a higher incidence of uropathogenic bacteria *Proteus* and a lower incidence of *Lactobacillus* in the urine of OAB patients [21]. Most patients were OAB wet. A reduced frequency of *Lactobacillus* occurrence in the urobiome was also observed in patients with UUI and MUI [17,19]. Once again, it appears that the genus *Lactobacillus* may have a protective role and be associated with the absence of urinary symptoms. Similar results to Curtiss et al. were obtained by Wu et al. In research comparing 30 women with OAB with 25 controls, in which urine was collected by TC, they found overrepresentation of seven genera (*Sneathia*, *Staphylococcus*, *Proteus*, *Helcococcus*, *Gemella*, *Mycoplasma* and *Aerococcus*) and underrepresentation of thirteen genera (*Prevotella*, *Dialister*, *Fusobacterium*, *Jonquetella*, *Campylobacter*, *Finegoldia*, *Anaerococcus*, *Lactobacillus*, *Pyramidobacter*, *Ureaplasma*, *Enterococcus*, *Novosphingobium* and *Lactococcus*) in the OAB group [22].

It seems that urinary microbiomes not only differ between OAB and healthy women, but also between OAB patients with a different severity of symptoms. Li et al. assessed 70 women with OAB who were divided into two groups by the severity of their symptoms, according to the overactive bladder symptom score (OABSS) questionnaire. Urine samples were obtained by TC. The study demonstrated that the urinary microbiome was associated with the severity of OAB, with greater diversity and richness of the urobiomes in the group with more severe OAB symptoms. In addition, certain bacteria genera were associated with specific symptoms of OAB, e.g., the abundance of *Prevotella* and *Porphyromonas* was positively correlated with the degree of nocturia [58].

The pathogenesis of OAB is multifactorial and is often associated with psychological disorders. In their work, Wu et al. assessed the relationship between the composition of the urinary microbiome and psychological factors. They found that the diversity of the urobiome was lower in depression cases compared to non-depression cases. Moreover, some bacterial genera of OAB patients with anxiety and depression were significantly different from those without [22].

The relationship of the microbiome to OAB opens the way to new treatments focused on modifying the microbiome. Thomas-White et al. assessed the effect of vaginal estrogen therapy on the urinary and vaginal microbiomes. Catheterized urine and voided urine, collected from 41 women with OAB who used 0.5 grams of estrogen cream vaginally twice a week for 12 weeks, were analyzed. The therapy resulted in a significant increase in *Lactobacillus* in the bladder, with no changes in the vagina or voided urine. The change in the amount of *Lactobacillus* in the bladder was associated with slight changes in urgency incontinence symptoms. However, bacterial identification was obtained by expanded quantitative urine culture, not by 16S rRNA gene sequencing [59]. Interestingly, the authors did not observe the expected decrease in *Lactobacillus* in the vagina. The authors suggest that this may be due to the short duration of the study. Further studies of longer duration are necessary to determine whether long-term vaginal estrogen therapy changes the vaginal microbiome, and whether changes in the urobiome result directly from the action of estrogen or are secondary to changes in the vaginal microbiome.

## 8. Neuropathic Bladder (NB)

Neuropathic bladder (NB) is a dysfunction of the lower urinary tract caused by damage or disease of the nervous system, resulting in impaired bladder function, in both storage and emptying [60].

A summary of the composition of the urinary microbiome in NB is presented in Table 7.

Fouts et al. compared the urinary microbiomes of 27 patients with NB due to spinal cord injury (SCI) with 26 controls. Urine samples were collected using the patient’s usual method of bladder drainage (MSU, TC). The study found significant differences in the urobiomes of NB patients and controls. The urine of NB patients was dominated by *Klebsiella*, *Escherichia* and *Enterococcus*, while in the control group by *Lactobacillus*, *Corynebacterium*, *Staphylococcus*, *Streptococcus*, *Gardnerella*, *Prevotella* and *Veillonella* [4]. Comparable results were obtained by Groah et al., who compared the urinary microbiomes of 24 patients with NB related to spinal cord injury with 23 controls. Urine samples were also collected in the patient’s usual way. The study found that the urinary microbiomes of NB patients had a significantly greater representation of *Enterococcus faecalis*, *Proteus mirabilis*, *Klebsiella pneumoniae* and *Pseudomonas aeruginosa*. In the control group, the urine microbiomes had a greater representation of *Lactobacillus crispatus* (females) and *Staphylococcus haemolyticus* (males). In NB patients, there was no significant difference in the urine microbiomes, depending on the method of emptying the bladder (intermittent catheterization, voiding or suprapubic catheterization) [23].

Fouts et al. also assessed whether the urinary microbiome differs with the duration of NB. It was found that, up to 2 months after the spinal cord injury, the urinary microbiomes of NB patients did not differ from those of healthy controls. However, after 13 months, differences were visible—the urinary microbiome was dominated by *Enterococcus*, and almost devoid of *Lactobacillus*. It is believed that these changes may play a role in the increased risk of UTI in these patients [4].

Urinary microbiome studies are also conducted in the pediatric population. Forster et al. characterized the urinary microbiomes of 36 children with NB who had their urine collected by catheterization. The results obtained were similar to those of adults, with the most abundant bacteria being unspecified *Enterobacteriaceae*, *Klebsiella*, *Staphylococcus*, *Streptococcus* and *Enterococcus*. The comparison of catheterization methods revealed that *Staphylococcus* was predominant in the urobiomes of children who catheterized their urethra, and that *Enterobacteriaceae* was predominant in the urobiomes of children catheterized through Mitrofanoff. These results differ from those obtained in the adult population by Groah et al.; however, the number of pediatric patients was too small to draw deeper conclusions [23,61]. 

Catheterization, both with an indwelling catheter and clean intermittent catheterization (CIC), are methods of management in patients with neurogenic dysfunction of the lower urinary tract. The results of studies assessing the impact of catheterization on the urobiome are ambiguous. As mentioned, Groah et al. found no differences in the urinary microbiomes of NB patients depending on the method of emptying the bladder [23]. Forster et al. found that *Staphylococcus* dominated in the urobiomes of children catheterized through the urethra, and *Enterobacteriaceae* dominated in the urobiomes of children catheterized through Mitrofanoff. However, this was an analysis of the pediatric population [61]. Lane et al. compared the urine microbiomes of patients with neurogenic lower urinary tract dysfunction who required catheterization. They compared 76 patients emptying their bladder by CIC with 18 patients requiring an indwelling catheter. They found that alpha diversity was lower in the CIC group, with no difference in beta diversity. In both groups, *Enterobacteriaceae* was the most common. In the indwelling catheter group, the genus *Pseudomonas* was more abundant compared to the CIC group, but the total abundance of *Pseudomonas* was low. However, the authors did not include patients who did not require catheterization in the analysis [62]. The number of studies assessing the impact of catheterization on the urinary microbiomes in patients with NB is very small, and the analyzed groups are very heterogeneous, which makes their comparison impossible; therefore, further research is necessary.

Changes in the microbiome during the use of probiotics can be used to assess the effectiveness and safety of UTI prophylaxis. Forster et al. assessed the safety and tolerability of a single intravesical *Lactobacillus rhamnosus* instillation in patients with NB. The study included five adults with SCI and five children with spina bifida. Urine was collected by clean intermittent catheterization before instillation and 7–10 days after. The authors demonstrated a significant difference in the beta diversity of the urobiomes of children and adults, both before and after instillation. These results are inconsistent with the results of the same author described above. There was no difference in the alpha diversity between the urinary microbiomes of both groups before and after instillation. Although most of the bacteria identified were present in the urinary microbiome before and after instillation, in the majority of cases the proportion of specific bacteria in the urobiome changed significantly after the instillation [61,63].

Bossa et al. assessed the change in the urinary microbiomes in three catheterized patients with SCI-related NB, depending on the use of prophylaxis or UTI development. The biofilm covering the tip of the catheter was analyzed. During the study, patients used oral probiotics (*Lactobacillus rhamnosus* with *Lactobacillus reuteri* and *Lactobacillus rhamnosus* with *Bifidobacterium*) for 6 months. The urinary microbiomes differed significantly between patients. The use of prophylaxis or the development of symptomatic UTIs led to a significant change in the composition of the catheter microbiomes, but these changes were transient, and the composition of the microbiomes returned to the pre-treatment state [64].

There are many neurological diseases in which lower urinary tract symptoms develop, e.g., multiple sclerosis, Parkinson’s disease or stroke. Currently, no studies have been published assessing the role of the urinary microbiome in these diseases. These are directions for potential further research.

## 9. Bladder Pain Syndrome/Painful Bladder Syndrome/Interstitial Cystitis (BPS)

According to the EAU guidelines, bladder pain syndrome (BPS) is defined as the occurrence of persistent or recurrent pain perceived in the urinary bladder region, accompanied by at least one other symptom, such as pain worsening with bladder filling and day-time and/or night-time urinary frequency. Moreover, there is no proven infection or other obvious local pathology. This disorder was formerly referred to as interstitial cystitis or painful bladder syndrome; however, the use of these terms is no longer recommended [65].

The etiology of this disease is multifactorial and not fully understood. In BPS, the urinary tract was considered sterile without clinical infection. However, due to the refutation of the urinary sterility dogma, the urinary microbiome is now considered in the pathogenesis of BPS.

A summary of the composition of the urinary s in BPS is presented in Table 8.

The first relevant studies were published as early as 1995, in which Domingue et al. demonstrated the presence of the bacterial 16S rRNA genes of Gram-negative bacteria in bladder biopsies from 29% of BPS patients, but not from the control group [69]. Two years later, Heritz et al. demonstrated the presence of bacterial genetic material in urine and bladder biopsy specimens in both BPS patients and controls, with no significant differences in the frequency of positive results between the groups. Urine and biopsy specimen cultures were negative in both groups [70]. 

The results of subsequent studies suggested that the urinary microbiome may play a role in the pathogenesis of BPS. A study comparing the microbiome of MSU from eight BPS patients with data obtained in a previous study of healthy patients by Siddiqui et el. found a reduced diversity and richness of the urinary microbiomes with a higher incidence of bacterial genus *Lactobacillus* in the BPS group compared to the control group (97% of sequence reads versus 57%). In addition, four genera (*Enterococcus*, *Atopobium, Proteus and Cronobacter*) were identified only in the urine of PBS patients. A significant increase in the incidence of *Lactobacillus* in the urine of BPS patients suggests a structural shift in the microbiomes in these patients [24]. Abernethy et al. compared urine samples collected by catheterization from 13 BPS patients and 18 controls. Similar to Siddiqui et al., they found less microbiome diversity and less distinct operational taxonomic units in the urine of PBS patients compared to the controls. However, the results regarding *Lactobacillus* did not repeat. The urine of patients with BPS was less likely to contain *Lactobacillus*, in particular *Lactobacillus acidophilus*. The presence of the bacterial genus *Lactobacillus* was associated with less severe symptoms, according to lower scores in the Interstitial Cystitis Symptoms Index and the Genitourinary Pain Index. Based on this, the authors suspected the protective role of a *Lactobacillus*-dominated, more diverse urobiome [25]. 

Despite promising results, more recent works by Bresler et al. [71], Meriwether et al. [72], Xu et al. [26], Jacobs et al. [73] and Nickel et al. [74] found no differences between the urinary microbiomes of BPS patients and controls. The authors analyzed both MSU samples and urine collected by catheterization. In most studies, the genus *Lactobacillus* was dominant in both the PBS and control groups [71,72,73,74], and only in one study by Xu et al. was *Lactobacillus* statistically less frequent in the BPS group. In the same work, four opportunistic types of pathogens (*Serratia*, *Brevibacterium*, *Porphyromonas and Citrobacter*) were statistically upregulated in BPS patients [26].

Jacobs et al. additionally assessed whether the urinary microbiome differs depending on the method of urine collection (MSU versus TC). They found that voided urine does not accurately reflect the bladder microbiome, and that its use to characterize the bladder microbiome could lead to a significantly elevated false positive rate. However, they used an extended quantitative urine culture for evaluation, and only used 16S rRNA gene sequencing in a subset as a control for the collection technique [73]. 

BPS patients often experience exacerbations of pain, known as flares. The cause was considered to be an infection, but these patients usually have sterile urine cultures, hence there is currently no evidence to support the hypothesis of a bacterial etiology of flares. Due to the limitations of standard urine culture, it is suspected that changes in the composition of the urine microbiome may be responsible for exacerbations of symptoms in BPS patients. Therefore, Nikcel et al. analyzed the urobiomes of 213 patients with PBS with negative standard urine culture. They compared the urine microbiomes of 127 patients who did not report flares with the urine microbiomes of 86 patients who reported flares. The authors defined flares as “symptoms that are much worse than usual”. Overall, the species composition did not significantly differ between flare and non-flare cases at any level, in a multivariate analysis for richness. However, a significantly higher prevalence of fungi, especially the genera *Candida* and *Saccharomyces*, was found in the flare group. This may indicate a potential role of fungi in the pathogenesis of exacerbations, but further research is necessary [66].

To date, only one study has evaluated the bladder microbiomes in patients with Hunner lesions in the course of BPS. Nickel et al. compared MSU from 59 patients with BPS, including 29 with Hunner lesions and 30 without. There were no significant differences in species abundance between groups, or in subgroups of women with and without Hunner lesions. However, the abundance of four species differed significantly in the male subgroups with and without Hunner lesions. *Negativicoccus succinivorans*, *Porphyromonas somerae* and *Mobiluncus curtisii* were more abundant in the urine of men with Hunner lesions, and *Corynebacterium renale* was more abundant in the urine of men without Hunner lesions. Additionally, they found significantly higher diversity in men with Hunner lesions compared to females with Hunner lesions, but no significant difference in diversity between patients with and without Hunner lesions overall [67]. Due to the availability of results from only one study, further research is needed.

Researchers are also focusing on changes in the microbiomes of BPS patients due to various treatments. In their work, Zhang et al. assessed whether nanobacteria were present in the urine and bladder biopsies of BPS patients, and whether tetracycline therapy reduced the level of nanobacteria and symptoms. Bladder biopsy nanobacteria was detected in 11 of the 27 assessed BPS patients. After combined oral and intravesical tetracycline therapy, all patients experienced a significant decrease in the level of nanobacteria and a statistically significant reduction in BPS symptoms [75].

In PBS therapy, effectiveness has also been demonstrated by non-pharmacological forms of therapy, such as mindfulness-based stress reduction. Shatakin-Margolis et al. assessed the effect of mindfulness-based stress reduction therapy on the urinary microbiomes of BPS patients. Urine collected by catheterization from 12 patients with BPS, before and after an 8-week mindfulness-based stress reduction course, was analyzed. The therapy included yoga and meditation once a week for 1 h. The study found an increase in diversity and a significant change in the composition of the urinary microbiomes after treatment. There was a relative increase in *Streptococcus* and *Porphyromonas* and a relative decrease in *Lactobacillus*. Notwithstanding the slight decrease in the amount of *Lactobacillus*, this bacterial genus was dominant in urine both before and after therapy. All patients experienced a significant reduction in symptoms after treatment [68]. The obtained results refer to those demonstrated by Siddiqui et al. described above [24]. Some authors, like Abernethy et al., suggest a protective role for the diversity of the microbiome [66]. However, the results may have been influenced by the use of intravesical instillation by the evaluated patients, half of whom continued the instillations during therapy, with a maximum of two instillations during the mindfulness-based stress reduction course. More research is needed to eliminate this impact [68]. 

In conclusion, despite numerous studies, the role of the urinary microbiome in the pathogenesis of BPS remains unclear.

## 10. Future Directions

The results of the research conducted so far are ambiguous and do not allow the drawing of decisive conclusions. The quality of the evidence is low, mainly due to small sample sizes. Not only are the analyzed groups small, but there is often a difference in the size of the study and control groups. Moreover, the study groups are very heterogeneous in terms of gender, age and characteristics related to the disease, e.g., stage of bladder cancer. Moreover, the method of collecting urine for analysis is very diverse not only between studies, but also within a given study. All these factors may distort the obtained results.

A uniform method of urine collection seems to be quite a challenging issue. It seems simple to collect urine through the MSU, but there are many opinions that such urine is contaminated by the urethral microbiome. TC appears to be an alternative, but its use in healthy controls raises significant ethical concerns. There is no doubt that only one method should be used within one study.

Therefore, it is necessary to conduct multicenter studies. To make this possible, it is necessary to develop a unified protocol covering the method of urine collection, its volume and the method of storing and transporting the material, as well as precise details regarding the isolation of genetic material and its sequencing.

Accurate characterization of the urobiome in various urological diseases may allow for its extensive use in clinical practice in the future. Microbiome composition analysis can be used to identify risk factors for specific urological diseases or as a non-invasive marker for the diagnosis of certain disorders. Urobiome analysis can also be used as a prognostic marker of the course of the disease or response to a given treatment, or as a prognostic marker. In addition, modification of the microbiome through diet, probiotics or antibiotic therapy can be used to prevent the occurrence of a given disease, as a method of treatment or as a combination therapy, allowing for a better response to existing treatment. This will allow for a personalized approach to the patient.

In the light of the overthrow of the dogma regarding the sterility of urine from the urinary tract and intensive research on the urobiome, the terms used so far, such as “uropathogens”, “asymptomatic bacteriuria”, “significant battery” seem to be inadequate, and this terminology should be re-evaluated.

## 11. Conclusions

In conclusion, the urinary system has its own endogenous microbiome, and the dogma that urine is sterile has been overthrown. Disturbances in the composition of the microbiome can lead to the development of various urological diseases, including urinary bladder diseases. Further research on the urobiome is needed to allow for a microbiome-focused, personalized approach to the patient.

## Figures and Tables

**Table 1:** Summary of urinary microbiome composition in bladder diseases.

Disease	Main Bacterial Taxa *	References
**Healthy**	*Citrobacter*, *Corynebacterium*, *Enterococcus*, *Escherichia*, *Lactobacillus*, *Prevotella*, *Staphylococcus*, *Streptococcus*	[4,5,6]
**Bladder cancer**	*Achromobacter*, *Acinetobacter*, *Actinobacteria*, *Actinobaculum*, *Actinomyces*, *Anaerococcus*, *Bacteroidete*, *Brevibacterium*, *Brucella*, *Campylobacter*, *Facklamia*, *Firmucutes*, *Fusobacterium*, *Proteobacteria*, *Pseudomonas*, *Sphingobacterium*, *Streptococcus*, *Tenericutes*	[8,9,10,11,12]
**Acute cystitis**	*Acinetobacter*, *Actinobacteria*, *Bacteroidetes*, *Enterobacteriaceae*, *Enterococcaceae*, *Firmicutes*, *Gammaproteobacteria*, *Proteobacteria*, *Pseudomonadaceae*, *Pseudomonas*	[13,14]
**Recurrent urinary tract infections**	*Bacteroidales*, *Bacteroidia*, *Bacteroidetes*, *Burkholderiales*, *Corynebacterium*, *Dialister*, *Firmicutes*, *Prevotella*, *Prevotellaceae*, *Proteobacteria*	[14,15]
**Urgency urinary incontinence**	*Actinobaculum*, *Actinomyces*, *Aerococcus*, *Arthrobacter*, *Corynebacterium*, *Gardnerella*, *Lactobacillus*, *Lactbacillus gasseri*, *Oligella*, *Staphylococcus*, *Streptococcus*	[16,17]
**Stress urinary incontinence**	*Corynebacterium*, *Enterobacter*, *Gardnerella*, *Lactobacillus*, *Staphylococcus*	[18]
**Mixed urinary incontinence**	*Escherichia*, *Gardnerella*, *Lactobacillus*, *Prevotella*, *Streptococcus*, *Tepidimonas*	[19,20]
**Overactive bladder**	*Aerococcus*, *Anaerococcus*, *Campylobacter*, *Dialister*, *Enterococcus*, *Finegoldia*, *Fusobacterium*, *Gemella*, *Helcococcus*, *Jonquetella*, *Lactobacillus*, *Lactococcus*, *Mycoplasma*, *Novosphingobium*, *Prevotella*, *Proteus*, *Pyramidobacter*, *Sneathia*, *Staphylococcus*, *Ureaplasma*	[21,22]
**Neuropathic bladder**	*Enterococcus*, *Escherichia*, *Klebsiella*, *Proteus*, *Pseudomonas*	[4,23]
**Bladder pain syndrome**	*Atopobium*, *Brevibacterium*, *Citrobacter*, *Cronobacter*, *Enterococcus*, *Lactobacillus*, *Porphyromonas*, *Proteus*, *Serratia*	[24,25,26]

* taxa have been arranged alphabetically.

**Table 2:** Summary of urinary microbiome composition in bladder cancer.

Patients’ Characteristics	Main Bacterial Taxa *	References
**Overall BC patients**	*Achromobacter*, *Acinetobacter*, *Actinobacteria*, *Actinobaculum*, *Actinomyces*, *Anaerococcus*, *Bacteroidete*, *Brevibacterium*, *Brucella*, *Campylobacter*, *Facklamia*, *Firmucutes*, *Fusobacterium*, *Proteobacteria*, *Pseudomonas*, *Sphingobacterium*, *Streptococcus*, *Tenericutes*	[8,9,10,11,12]
**Females**	*Actinotignum*, *Campylobacter*, *Klebsiella*, *Lactobacillus*, *Prevotella*, *Vellionella*	[10,28]
**Smokers**	*Bacteroidaceae*, *Bacteroides*, *Erysipelotrichales*, *Lachnospiraceae*	[29]
**Recurrent BC**	*Acinetobacter*, *Anoxybacillus*, *Brachybacterium*, *Corynebacterium*, *Geomicrobium*, *Jeotgalibacillus*, *Larkinella*, *Massilia*, *Micrococcus*, *Nocardioides*, *Pseudomonas*, *Staphylococcus*, *Thermomonas*	[30,31]
High risk of progression **	*Bacteroides*, *Herbaspirillum*, *Marmoricola*, *Porphyrobacter*	[12]
High risk of recurrence **	*Aeromonas*, *Bacteroides*, *Faecalibacterium*, *Gemella*, *Herbaspirillum*, *Porphyrobacter*	[12]
**NMIBC**	*Cupriavidus*	[10]
**MIBC**	*Bacteroides*, *Faecalbacterium*, *Hemophilus*, *Veilonella*	[10,32]
**PD-L1-positive patients**	*Leptotrichia*, *Propionibacteriu*, *Roseomonas*	[33]
**BC tissue**	*Acinetobacter*, *Actinobacteria*, *Akkermansia*, *Anoxybacillus*, *Bacteroides*, *Bacteroidetes*, *Brucellaceae*, *Clostridium*, *Cupriavidus*, *Enterobacter*, *Enterococcus*, *Escherichia-Shigella*, *Firmicutes*, *Geobacillus*, *Klebsiella*, *Pelomonas*, *Proteobacteria*, *Ralstonia*, *Sphingomonas*, *Staphylococcus*	[34,35,36]
**BCG-therapy responders**	*Brochothrix*, *Escherichia-Shigella*, *Firmicutes*, *Lactobacillales*, *Negativicoccus*, *Pseudomonas*, *Serratia*	[10,37]
**BCG-therapy nonresponders**	*Gammaproteobacteria*, *Proteobacteria*	[37]

BC—bladder cancer, NMIBC—non-muscle-invasive BC, MIBC—muscle-invasive BC, PD-L1—programmed cell death protein ligand 1, BCG—Bacillus Calmette-Guérin; * taxa have been arranged alphabetically; ** based on the EORCT score model.

**Table 3:** Summary of urinary microbiome composition in urinary tract infections.

Patients’ Characteristics	Main Bacterial Taxa *	References
**Acute cystitis**		
**Overall AC patients**	*Acinetobacter*, *Actinobacteria*, *Bacteroidetes*, *Enterobacteriaceae*, *Enterococcaceae*, *Firmicutes*, *Gammaproteobacteria*, *Proteobacteria*, *Pseudomonadaceae*, *Pseudomonas*	[13,14]
**Younger patients**	*Enterobacteriaceae*, in particular *E. coli*	[42]
**Older** **patients**	*Firmicutes*, *Pseudomonas*	[42]
**Females**	*Enterobacteriaceae*, *Lactobacillus*, *Staphylococcus*, *Streptococcus*	[42]
**Recurrent urinary tract infection**		
**Overall rUTI patients**	*Bacteroidales*, *Bacteroidia*, *Bacteroidetes*, *Burkholderiales*, *Corynebacterium*, *Dialister*, *Firmicutes*, *Prevotella*, *Prevotellaceae*, *Proteobacteria*, *Ralstonia*, *Rothia*, *Sphingomonas*, *Staphylococcus*, *Streptococcus*	[14,15]
**Topical estrogen therapy**	*Lactobacillus crispatus*	[43]

AC—acute cystitis, rUTI—recurrent urinary tract infection; * taxa have been arranged alphabetically.

**Table 4:** Summary of urinary microbiome composition in urinary incontinence.

Patients’ Characteristics	Main Bacterial Taxa *	References
**Urgency urinary incontinence**		
**Overall UUI patients**	*Actinobaculum*, *Actinomyces*, *Aerococcus*, *Arthrobacter*, *Corynebacterium*, *Gardnerella*, *Lactobacillus*, *Lactbacillus gasseri*, *Oligella*, *Staphylococcus*, *Streptococcus*	[16,17]
**Younger,** **↑ BMI**, ***↑* urge episodes, ↓ UTI, better treatment response**	*Aerococcus*, *Bifidobacterium*, *Enterobacteriaceae*, *Gardnerella*, *Lactobacillus*, *Prevotella*, *Staphylococcus*	[51]
**Stress urinary incontinence**		
**Overall SUI patients**	*Corynebacterium*, *Enterobacter*, *Gardnerella*, *Lactobacillus*, *Staphylococcus*	[18]
**Hormone-positive patients**	*Gardnerella*, *Lactobacillus*	[52]
**Mixed Urinary Incontinence**		
**Overall MUI patients**	*Escherichia*, *Gardnerella*, *Lactobacillus*, *Prevotella*, *Streptococcus*, *Tepidimonas*	[19,20]

UUI—urgency urinary incontinence, SUI—stress urinary incontinence, MUI—Mixed Urinary Incontinence, ↑—increase, ↓—decrease; * taxa have been arranged alphabetically.

**Table 5:** Summary of urinary microbiome composition in urgency urinary incontinence.

Microbiome Composition Compared to Healthy Controls *				References
No differences in diversity and richness				[16,17,53]
*↑ Gardnerella*, *↓ Lactobacillus*				[17]
*Lactbacillus gasseri*—more common in the UUI group *Lactobacillus crispatus*—more common in controls				[17]
*Actinobaculum*, *Actinomyces*, *Aerococcus*, *Arthrobacter*, *Corynebacterium*, *Gardnerella*, *Oligella*, *Staphylococcus*, *Streptococcus*—more common in UUI group				[17]
*Actinobaculum*, *Aerococcus*, *Arthrobacter*, *Oligella*—isolated only in UUI patients				[17]
**Microbiome composition changes depending on the treatment method**				
**Antimuscarinic therapy**	**Mirabegron**	**Resistance to antimuscarinic therapy**	**Sacral neuromodulation**	
Diversity: Responders < Nonresponders	Diversity: Responders > Nonresponders	↑ diversity	No differences	

UUI—urgency urinary incontinence, ↑—increase, ↓—decrease; * taxa have been arranged alphabetically.

**Table 6:** Summary of urinary microbiome composition in overactive bladder.

Patients’ Characteristics	Main Bacterial Taxa *	References
**Overall OAB patients**	*Aerococcus*, *Anaerococcus*, *Campylobacter*, *Dialister*, *Enterococcus*, *Finegoldia*, *Fusobacterium*, *Gemella*, *Helcococcus*, *Jonquetella*, *Lactobacillus*, *Lactococcus*, *Mycoplasma*, *Novosphingobium*, *Prevotella*, *Proteus*, *Pyramidobacter*, *Sneathia*, *Staphylococcus*, *Ureaplasma*	[21,22]
**OAB patients with nocturia**	*Porphyromona*, *Prevotella*	[58]

OAB—overactive bladder; * taxa have been arranged alphabetically.

**Table 7:** Summary of urinary microbiome composition in neuropathic bladder.

Patients’ Characteristics	Main Bacterial Taxa *	References
**Overall NB patients**	*Enterococcus*, *Escherichia*, *Klebsiella*, *Proteus*, *Pseudomonas*	[4,23]
**Children**	*Enterobacteriaceae*, *Enterococcus*, *Klebsiella*, *Staphylococcus*, *Streptococcus*	[61]
**Children catheterizing the urethra**	*Staphylococcus*	[61]
**Children catheterizing through Mitrofanoff**	*Enterobacteriaceae*	[61]
**Adults requiring catheterization**	*Enterobacteriaceae*	[62]
**Adults requiring indwelling catheter**	*Pseudomonas*	[62]

NB—neuropathic bladder; * taxa have been arranged alphabetically.

**Table 8:** Summary of urinary microbiome composition in bladder pain syndrome.

Patients’ Characteristics	Main Bacterial Taxa *	References
**Overall BPS patients**	*Atopobium*, *Brevibacterium*, *Citrobacter*, *Cronobacter*, *Enterococcus*, *Lactobacillus*, *Porphyromonas*, *Proteus*, *Serratia*	[24,25,26]
**BPS patients with flares**	*Candida*, *Saccharomyces*	[66]
**Male BPS patients with Hunner lesions**	*Mobiluncus curtisii*, *Negativicoccus succinivorans*, *Porphyromonas somerae*	[67]
**Male BPS patients without Hunner lesions**	*Corynebacterium renale*	[67]
**BPS patients after mindfulness-based stress reduction therapy**	*Lactobacillus, Porphyromonas, Streptococcus*	[68]

BPS—bladder pain syndrome; * taxa have been arranged alphabetically.

## Data Availability

No new data were created or analyzed in this study. Data sharing is not applicable to this article.
